# Risk factors for recurrent wheezing – international study of wheezing in infants (EISL) phase 3

**DOI:** 10.1186/1939-4551-8-S1-A123

**Published:** 2015-04-08

**Authors:** Carolina Aranda, Gustavo Falbo Wandalsen, Dirceu Sole, Ligia Fonzar, Ana Caroline Dela Bianca, Javier Mallol

**Affiliations:** 1 Hospital Do Servidor Publico Municipal De São Paulo, Brazil; 2 Federal University of Sao Paulo, Brazil; 3 Brazilian Society, Brazil; 4 Universidade Faderal De Pernambuco, Brazil; 5 University of Santiago De Chile (USACH), Chile

## Background

We aimed to identify factors associated with recurrent wheezing (RW) in infants in the first year of life living in the Southern region of São Paulo city and participating in the “*Estudio Internacional de Sibilancias en Lactantes* (EISL)” – phase 3 (P3).

## Methods

Parents of infants who were attended in primary care health units in the Southern region of São Paulo city from 2009 to 2010 answered the EISL-P3 written questionnaire. The wheezing group was stratified in accordance to the frequency of wheezing episodes as occasional wheezing (OW, less than three episodes), or RW (three or more episodes). Wheezing-associated factors were evaluated using multivariate analysis and were expressed as odds ratio (OR) and 95% confidence interval (95%CI).

## Results

The most relevant factors related to OW were pneumonia (OR=3.10, 95%CI=1.68-5.73), hospitalization due to pneumonia (OR=2.88, 95%CI=1.26-6.56) and recurrent upper respiratory infection (URI, OR=1.87, 95%CI=1.25-2.81). Regarding RW, recurrent URI (OR=5.34, 95%CI=3.83-7.45), pneumonia (OR=4.06, 95%CI=2.87-5.74) and asthmatic siblings (OR=3.02, 95%CI=1.67-5.45) were the most significantly associated factors.

## Conclusions

In the present study, we found that recurrent URI, positive history of pneumonia and familiar history of asthma were the most relevant factors associated with RW. The precocious knowledge of these factors can enable the identification of the probable asthmatic infants and can improve both prevention strategies and treatment of these patients.

